# Medical and Philosophical News

**Published:** 1784

**Authors:** 


### SECTION III.

### Medical and Philosophical News.

THE Medical Society of London, held in Crane-court, Fleet-street, have proposed the following question, as a subject for a prize medal of ten guineas value, viz. "What diseases "may be mitigated or cured, by exciting par- "ticular affections or passions of the mind?" Dissertations on this subject must be written in Latin, English, or French, and delivered to the secretary, at least two months before the meeting for adjudging the medal, which will be on the 8th day of March, 1786.

The

The Royal Medical Society at Paris, not being ſufficiently ſatisfied with any of the diſſertations that have been ſent to them on the prize queſtion announced laſt year, (*ſee vol.* IV. *page* 204) relative to the connection between the liver and the ſkin, have agreed to propoſe it a ſecond time.—Diſſertations on this ſubject muſt be ſent to M. Vicq d'Azyr, ſecretary of the ſociety, before the 1ſt of May, 1786.

The ſociety offer a premium of 300 livres to the perſon who ſhall "aſcertain the advantages "which the practice of phyſic may be able to "derive from the modern diſcoveries relative "to the art of determining the purity of the "air by different eudiometers."—Diſſertations on this ſubject will be received by the ſecretary till the 1ſt of July, 1785.

The King has been pleaſed to incorporate the ſurgeons of Dublin into a college, to be called the Royal College of Surgeons of Ireland, with authority to examine and grant letters teſtimonial to all ſuch perſons as ſhall be deemed qualified to practice ſurgery in that kingdom. The preſent officers of the college are,

| | | |
|---|---|---|
| Samuel Croker King, Eſq. | | *Preſident.* |
| Mr. John Whiteway, | } | *Cenſors.* |
| Mr. Henry Lyſter, | | |
| Mr. Robert Bowes, | | |
| Mr. Guſtavus Hume, | | |
| Mr. Philip Woodroffe, | | |
| Mr. James Henthorn, | | *Secretary.* |

---

Extract of a letter from Dr. Houlſton, of Liverpool, to Dr. Simmons; dated Aug. 27, 1784.

"Within the town of Wigan, in Lancaſhire, there has lately been diſcovered, in boring for coal, a ſulphureous ſpring, reſembling in its qualities the *Harrogate water*. It riſes in large quantity, is perfectly clear and tranſparent, and is not very offenſive either to the ſmell or taſte; —much leſs ſo than the Harrogate water; this being not nearly ſo ſtrongly impregnated with the ſulphureous principle. If carefully corked, it will remain for ſome time unimpaired, but expoſed to the air, it ſoon loſes much of its ſmell and efficacy.

"It

"It is already greatly reſorted to, and is thought to have produced remarkable good effects in many caſes, particularly in cutaneous and ſcorbutic eruptions, to which it ſeems peculiarly adapted.——The proprietor of it is making a reſervoir, and taking other ſteps to render the acceſs to it more commodious; and there is ſaid to be an intention of building baths, by which, the advantages to be expected from its uſe may be conſiderably increaſed."

---

Extract of a letter from Mr. Edward Jacob, junior, ſurgeon at Faverſham in Kent, to Dr. Simmons; dated Auguſt 30, 1784.

"I had lately an opportunity of giving relief to a poor man, in a caſe which, without timely aſſiſtance, might have been a cauſe of pain and uneaſineſs all his life time. It was a clear and perfect luxation of the head of the os femoris. At firſt I ſuſpected there might be a fracture at the head of the bone, being aware, that on account of the great degree of violence neceſſary to produce a luxation of the thigh bone, ſome have even doubted the poſſibility of ſuch an an accident. But being ſoon clearly convinced of the nature of the caſe, I proceeded to the reduction, which was effected in a very ſhort time by a ſteady extenſion and counter extenſion. The noiſe produced by the return of the bone into its ſocket, was diſtinctly heard by me and my aſſiſtants; and immediately after the reduction, the patient, who before had been unable to move his leg, was able to raiſe it without help. The next day he walked down ſtairs with the aſſiſtance of crutches; and in a few days after the accident, went about his ordinary buſineſs. On peruſing the 2d vol. of *Med. Obſ.* and *Inq.* I find a ſimilar caſe mentioned by Mr. Travis, which tempts me to write this to you."

---

The ſection of the ſymphyſis pubis has lately been performed at Paris, for the fourth time (in that metropolis), with ſucceſs. The patient was a ricketty woman, twenty-nine years old, who had twice before been pregnant, and had each time been delivered, with difficulty, of a dead child. The operation of the ſection, in this third labour, was performed by M. de Matthiis, in the preſence of M. Alphonſo le Roy and others; others; and the patient was delivered of a live child. When this account of the caſe was written, (Auguſt 7, the day after the operation) the ſtate of the patient was ſaid to be extremely favourable.

---

In a former part of this volume (*page* 85) we had occaſion to mention an inſtance of hydrophobia. Another melancholy caſe of the ſame kind has lately occurred at New Grange, near Bolton upon Dearne in Yorkſhire. The patient, a poor labouring man, named George Wilſon, was bit in one of his hands by a mad dog, at the beginning of May laſt, and having taken ſome pretended remedy, which had been recommended to him by one of his neighbours, thought himſelf ſecure. On Wedneſday, September the 8th, ten weeks after the accident, he was ſeized about noon with ſymptoms of hydrophobia, and died in the evening of the Friday following.

---

Mr. James Watt has lately communicated to the Royal Society, a new method of preparing a teſt a test liquor to shew the presence of acids and alkalis in chemical mixtures.—Not being satisfied with the usual tests of infusions of tournesol and litmus for ascertaining the point of saturation of acids and alkalis, Mr. Watt tried several vegetable juices, and among them found that the syrup of red cabbage (*brassica rubra*) furnished the best test, and in its fresh state has more sensibility both to acids and alkalis, than any other before used.

---

Mr. Martineau, surgeon at Norwich, has lately communicated to the Royal Society, a remarkable case of dropsy of the ovarium. The disease began to appear after a miscarriage, when the patient was in her twenty-seventh year. She first underwent the operation of tapping in 1757, and afterwards had recourse to it three, four, or five times a year, till her death, which happened in 1783. In that space of time, she was tapped 80 times, and lost 6631 pints of fluid.—On dissection, the left ovarium was found enlarged into an immense pouch. The peritonæum was thickened, and in some places ossified; a proof, according to Mr. Martineau, of the provision occasionally made by nature for the preſervation of animals, by thus ſecuring parts, which, if ſuddenly rent, would be fatal, whereas collections of matter are always allowed to make their way out of the body.

---

*New works about to be publiſhed.*—1. An Inquiry into the various theories and different methods of cure in Apoplexies and Palſies; by Dr. Chandler, Phyſician at Canterbury.—2. An Inquiry how to prevent the Small Pox; to which will be added, an Account of the Proceedings of a Society for promoting general Inoculation at ſtated periods, and preventing the natural Small Pox in Cheſter: by Dr. Haygarth, F.R.S.—3. A large Volume in folio, in Engliſh and Latin, entitled, the Natural Hiſtory and Pathology of Fiſhes, with many copper plates; by Dr. Monro, Profeſſor of Anatomy at Edinburgh.

